# Population-Wide Emergence of Antiviral Resistance during Pandemic Influenza

**DOI:** 10.1371/journal.pone.0001839

**Published:** 2008-03-19

**Authors:** Seyed M. Moghadas, Christopher S. Bowman, Gergely Röst, Jianhong Wu

**Affiliations:** 1 Institute for Biodiagnostics, National Research Council Canada, Winnipeg, Manitoba, Canada; 2 Department of Mathematics and Statistics, The University of Winnipeg, Winnipeg, Manitoba, Canada; 3 Department of Electrical and Computer Engineering, The University of Manitoba, Winnipeg, Manitoba, Canada; 4 Analysis and Stochastics Research Group, Hungarian Academy of Sciences, Bolyai Institute, University of Szeged, Szeged, Hungary; 5 Department of Mathematics and Statistics, York University, Toronto, Ontario, Canada; 6 Centre for Disease Modelling, York Institute of Health Research, York University, Toronto, Ontario, Canada; Yale University, United States of America

## Abstract

**Background:**

The emergence of neuraminidase inhibitor resistance has raised concerns about the prudent use of antiviral drugs in response to the next influenza pandemic. While resistant strains may initially emerge with compromised viral fitness, mutations that largely compensate for this impaired fitness can arise. Understanding the extent to which these mutations affect the spread of disease in the population can have important implications for developing pandemic plans.

**Methodology/Principal Findings:**

By employing a deterministic mathematical model, we investigate possible scenarios for the emergence of population-wide resistance in the presence of antiviral drugs. The results show that if the treatment level (the fraction of clinical infections which receives treatment) is maintained constant during the course of the outbreak, there is an optimal level that minimizes the final size of the pandemic. However, aggressive treatment above the optimal level can substantially promote the spread of highly transmissible resistant mutants and increase the total number of infections. We demonstrate that resistant outbreaks can occur more readily when the spread of disease is further delayed by applying other curtailing measures, even if treatment levels are kept modest. However, by changing treatment levels over the course of the pandemic, it is possible to reduce the final size of the pandemic below the minimum achieved at the optimal constant level. This reduction can occur with low treatment levels during the early stages of the pandemic, followed by a sharp increase in drug-use before the virus becomes widely spread.

**Conclusions/Significance:**

Our findings suggest that an adaptive antiviral strategy with conservative initial treatment levels, followed by a timely increase in the scale of drug-use, can minimize the final size of a pandemic while preventing large outbreaks of resistant infections.

## Introduction

The use of antiviral drugs to mitigate the impact of a nascent influenza pandemic has been evaluated in several recent modelling studies [1]–[7], with significant public health implications for identifying effective preparedness strategies. These studies suggest that early diagnosis and prompt onset of treatment of clinical cases is crucial for possible containment of a pandemic. A key assumption is that the virus remains less transmissible than pandemic viruses of the last century, so that the reproduction number of disease transmission stays below 1.8 [1], [6], [7]. However, the effectiveness of antiviral drugs may be diminished by several factors, including a delay in start of treatment, and more importantly, the emergence and transmission of drug-resistant viral mutants in the population [8]–[12].

While antiviral therapy appears to be central in any containment strategy, it will impact the emergence of drug-resistance in a complex manner. On one hand, early application of antiviral drugs will largely inhibit generation of resistant viruses by suppressing viral replication. On the other hand, it results in a longer time for selection in favour of pre-existing resistant mutants to restore their impaired replication fitness through compensatory mutations [13], [14]. With sufficiently increased fitness, resistant viruses may gain a competitive advantage in the spread of infection and establish a self-sustaining epidemic of viral resistance [8], [9], [14]. Strategic use of antiviral drugs is therefore crucial for not only mitigating the impact of the wild-type strain, but also preventing the occurrence of pandemic waves of drug-resistant infections.

The dynamics of competition between the wild-type and resistant strains is in general complex. If treatment is poorly administered, then the wild-type strain spreads rapidly and depletes the pool of susceptibles in the population, which would afford little chance for resistant strains to evolve or cause an outbreak of drug-resistance [8], [9]. It has been suggested that intensive antiviral treatment may eliminate the wild-type infection (when transmission of the virus is largely interrupted) without promoting the spread of resistant strains if transmissibility of resistant strains is sufficiently low [9]. Regardless of the feasibility of such antiviral strategy, high treatment levels can exert strong selective pressures that confer resistance that frequently evolves far more rapidly than the natural rate. The evolution of such mutants is influenced by several factors, including the duration of treatment, the delay in onset of therapy, and the rate at which de novo resistant mutations occur [8]. Combined with compensatory mutations that raise fitness of resistant viruses [13], [14], intensive treatment may indeed result in a devastating pandemic of resistant viral mutants. Understanding the dynamics of the emergence of drug-resistance is therefore crucial for implementation of effective mitigation strategies.

In this paper, we extend previous work [8], [15] to illustrate the possible scenarios of disease outbreak in the population, including single-strain infections and co-existence of wild-type and resistant infections. By incorporating compensatory mutations into a mathematical model, we discuss the role of the transmission fitness of resistant mutants in determining the outcomes of antiviral strategies with constant and varying treatment levels. In the following, we describe the model based on the existing frameworks [8], [15], and provide details of the equations and analysis in “Text S1”. We derive the control reproduction number of the wild-type strain and use it for delineating the results and their epidemiological consequences for the strategic use of antiviral drugs in response to a future pandemic.

## Methods

### The model

**Figure 1:**
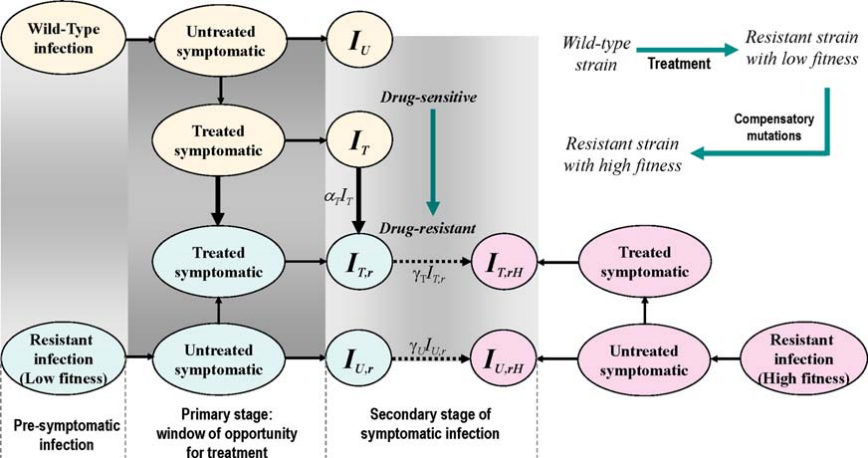
Model structure for the emergence of drug-resistance during treatment of symptomatic infections. The clinical course of disease is divided into three stages: pre-symptomatic; primary; and secondary stages of symptomatic infections. Drug-resistance with low transmission fitness can emerge during treatment of individuals infected with the wild-type virus. It is assumed that compensatory mutations may result in generation of resistant mutants with high transmission fitness during the secondary stage of treated symptomatic infection. The compartments of untreated and treated individuals infected with the wild-type strain are represented by *I_U_* and *I_T_*, respectively. The corresponding compartments for the resistant strain with low (high) transmission fitness are denoted by sub-index *r* (*rH*).

To develop a population dynamical model, we followed previous work [8], [15] and divided the population into several compartments comprising susceptible, exposed, asymptomatic, and symptomatic infected individuals. In our model, exposed individuals undergo a latent period, during which viral titers increase to detectable and transmissible levels [16]. An exposed individual may become infectious after the latent period and shed virus without showing clinical symptoms; this is referred to as asymptomatic infection. Considering the kinetics of influenza infection in humans [16], we divided the clinical course of infection into three stages: (i) pre-symptomatic infection, (ii) primary stage of symptomatic infection (referred to as the window of opportunity for start of treatment); and (iii) secondary stage of symptomatic infection (Figure 1). The relative transmissibility of the virus at each stage is estimated by superposing a step-function on the log-normal curve fitted to household longitudinal data on influenza viral shedding [1], [17]. Antiviral treatment, as a single containment strategy, may be initiated upon diagnosis of a clinical case within the window of opportunity; however, those who have not started treatment in this window will receive no antiviral therapy during the secondary stage of symptomatic infection. The probability of an individual receiving treatment decreases with the time elapsed since the onset of symptoms, which is reflected in the functional form of the treatment rate with delay in seeking healthcare [8], [15]. We assumed that treatment reduces the infectiousness level of the wild-type disease by 60% (reflected as a reduced transmission rate in the model since initiation of treatment), but has no effect on individuals infected with resistant viruses.

We considered a two-day window of opportunity for initiating treatment of indexed cases following the onset of clinical disease. It is assumed that resistant mutants with low transmission fitness (δ*_r_*) emerge during treatment of individuals infected with the wild-type strain. With continuous replication of the virus, the rate of developing de-novo resistance is greatest when treatment is started near the peak of viral titers [8]. Although resistant mutants may initially emerge with compromised fitness and growth [18], mutations that compensate for this impaired fitness may arise [13], [14]. These compensatory mutations can generate variants with high transmission fitness (δ*_rH_*), comparable to that of the wild-type strain. Such mutations are more likely to occur during the secondary stage of symptomatic infection, as resistant mutants in viruses isolated from treated patients were mostly detected 3 days after the onset of treatment [19], [20]. We extended the model to include compartments of individuals who are carriers of highly transmissible resistant viruses (either evolved during treatment or transmitted through direct person-to-person contacts) in both asymptomatic and symptomatic infection. We incorporated parameters for the treatment and emergence of drug-resistance (Table 1) into a deterministic epidemic model formulated by a system of delay differential equations (see “Text S1”).

**Table 1:** Description of transmission, mutation, and treatment parameters of the model with their baseline values and ranges used for simulations and sensitivity analyses.

Symbol	Description	Value (Range)	Reference
δ_*r*_	relative transmissibility of resistant strain with low fitness	0.2 (<0.4)	[8], [9], [11]
δ_*rH*_	relative transmissibility of resistant strain with high fitness	variable (>0.6)	[8], [9], [11]
ρ_max_	maximum rate of emergence of drug-resistance within the window of opportunity	0.018 (0.018–0.072) day^−1^	[11], [12]
α_*T*_	rate of emergence of drug-resistance during secondary stage of symptomatic infection	0.018 (0.018–0.072) day^−1^	[11], [12]
γ_*U*_	rate of conversion between resistant mutants in untreated symptomatic infection	3.6×10^−4^ (10^−6^–10^−1^) day^−1^	[14]
γ_*T*_	rate of conversion between resistant mutants in treated symptomatic infection	3.6×10^−3^ (10^−6^–10^−1^) day^−1^	[14]
1−*q*	fraction of infected individuals which receives treatment (treatment level)	variable (0–1)	−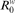	reproduction number of the wild-type strain	1.6 (1.4–2)	[1], [2], [4], [6], [7]

### Reproduction numbers

In order to evaluate the effect of parameters described in Table 1 on disease propagation, we calculated the control reproduction number of the wild-type strain (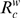), as a function of treatment level and delay in onset of therapy. In the absence of treatment, the quantity 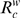 reduces to the basic reproduction number (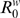), defined as the number of new infections generated by a single infected case introduced into a wholly susceptible population [21], and given by
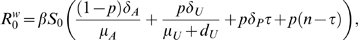
where β is the baseline transmission rate of the wild-type virus; *S*_0_ is the size of the susceptible population at the onset of pandemic; *p* represents the probability of developing clinical disease; μ*_A_* and μ*_U_* are, respectively, the recovery rate of asymptomatic and symptomatic infections (secondary stage); δ*_A_*, δ*_P_*, and δ*_U_* represent the relative transmissibility of the virus during asymptomatic, pre-symptomatic, and secondary stage of symptomatic infections; *d_U_* is the disease-induced death rate; τ is the period of pre-symptomatic infection; and *n* represents the period of the primary stage of symptomatic infection (see Table 1 in “Text S1”). We also derived the expressions for the number of new infections generated through direct transmission of resistant viruses with low fitness (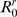) and high fitness (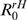), and obtained a criterion for the control of disease (see “Text S1”).

## Results

We considered the scenario in which a novel transmissible pandemic virus arises (at time *t* = 0) in a susceptible population of size *S*_0_ = 100 000 with no pre-existing immunity. We assumed that the treatment of indexed cases begins one day after the onset of clinical disease. The rate of de novo resistance (α*_T_*) that generates mutants with low fitness ranges from 0.018 to 0.072 day^−1^ [11], [12], and we assumed a baseline value of α*_T_* = 0.018 day^−1^. In our model, this rate results in the emergence of drug-resistance in approximately 4.8% of treated patients during secondary stage of symptomatic infection, with very marginal dependence on treatment level. We used the same rate for resistance emergence in the primary stage of symptomatic infection, which allows for the development of approximately 1% resistant infections during the primary stage of symptomatic infection. These rates contribute to an overall (approximately) 5.8% resistance emergence, which lies within the estimated range 1%–18% incidence of neuraminidase resistance reported in clinical samples [18], [19], [20].

We assumed that the fraction of treated individuals (hosting resistant viruses with low fitness) which undergoes compensatory mutations and subsequently generates resistant strains with high fitness lies between 1/5000 and 1/500 [9]. This is 10-fold greater than the corresponding fraction of untreated resistant cases [14]. We used these fractions to determine the ranges of conversion rates between low and high fitness resistant strains. To illustrate the typical outbreaks of wild-type and resistant infections, we inserted the following parameter values: γ*_T_* = 0.0036 day^−1^; γ*_U_* = 0.00036 day^−1^; δ*_r_* = 0.2; δ*_rH_* = 0.9; which correspond to probability 5×10^−4^ that a treated individual infected with the wild-type virus develops drug-resistance with high transmission fitness. Baseline values of these parameters and their respective ranges used for simulations and sensitivity analyses are given in Table 1, and details are provided in “Text S1”.

### Constant treatment strategy

**Figure 2:**
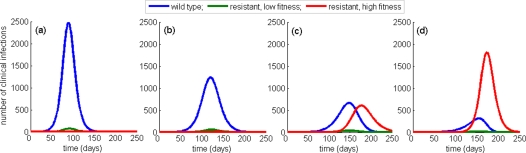
Time-courses of clinical infections with one day delay in onset of treatment of indexed cases, with 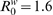. Simulations were run, when a single case infected with the wild-type virus is introduced into the susceptible population of size *S*_0_ = 100 000. Treatment levels are: (a) 50%; (b) 78%; (c) 90%; and (d) 95%. The corresponding reproduction numbers of the wild-type strain are: (a) 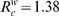; (b) 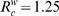; (c) 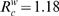; and (d) 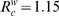.

Assuming 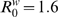 and 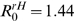, Figure 2 shows the occurrence of disease outbreaks for constant treatment levels during the entire course of the pandemic. For 50% treatment level of clinical cases, the wild-type strain spreads quickly and depletes the susceptible population, and therefore a limited number of resistant cases is generated (Figure 2a). Increasing treatment level to 78% leads to a reduction in the clinical attack rate of the wild-type virus from 22% (at 50% treatment level) to 16%, and lowers 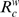 from 1.38 to 1.25 (Figure 2b). In this case, however, the emergent resistant mutants begin to invade the susceptible hosts and establish a self-sustaining epidemic. Further increase in the treatment level to 90% enhances the spread of resistant mutants and leads to the co-existence of outbreaks (Figure 2c). With higher treatment level (95%), 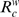 is reduced considerably below 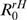, and the resistant outbreak substantially dominates that of the wild-type strain (Figure 2d). Transmission of wild-type infections is dramatically reduced, resulting in a 4% clinical attack rate. However, the wide-spread presence of resistant strains results in a higher overall attack rate than would have been the case if treatment were administered at a lower rate. We observed similar patterns for outbreaks of wild-type and resistant infections with higher values of 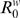. However, in these cases, wide-spread drug-resistance is less probable and requires higher levels of treatment to significantly interrupt the transmission of wild-type infections.

**Figure 3:**
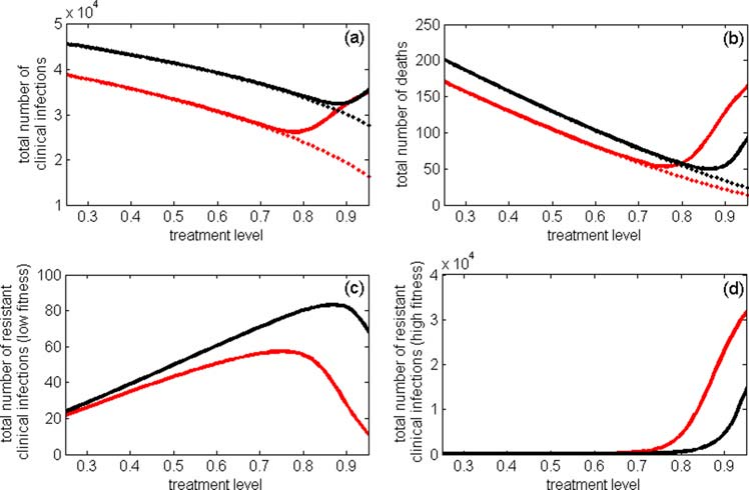
(a) Total number of clinical infections caused by the wild-type, low fitness resistant, and high fitness resistant strains; (b) Total number of deaths; (c) Total number of clinical infections caused by the low fitness resistant strains; (d) Total number of clinical infections caused by the high fitness resistant strains, as a function of treatment level. Treatment of infected individuals begins one day after the onset of clinical disease; red and black curves correspond, respectively, to the reproduction numbers 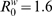 (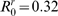, 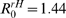) and 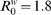 (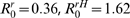). Dashed curves in (a) and (b) correspond to the scenario in which no compensatory mutations occur, and resistant mutants are only present at low fitness cost.

**Figure 4:**
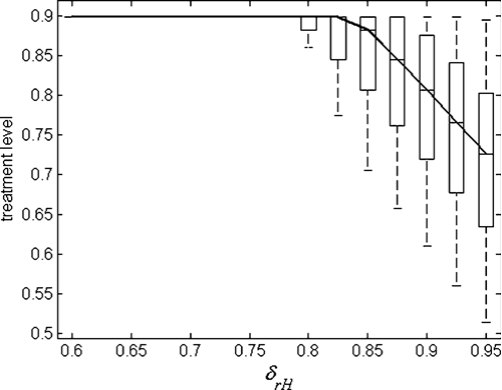
Sensitivity analysis showing box plots for the variations in the optimal constant treatment level (below 90%) as a function of δ*_rH_*, with other parameters sampled from their respective ranges, as described in “Text S1”. The solid curve passes through the median values of the treatment level, and each box contains 50% of data points between the first and third quartiles of the sampling distribution. The remaining 50% of data points are represented by whiskers.

Although the use of antiviral drugs appears to be essential for combating the wild-type strain, it can potentially lead to the population-wide spread of drug-resistance. To demonstrate the interplay between these opposing effects, we simulated the model to determine the final size of the epidemic, using 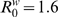 and 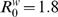, as a function of treatment level. The solid curves in Figures 3a–b show the total number of clinical infections and deaths during the entire course of an outbreak. As is evident, increasing the treatment level decreases the overall number of infections to a minimum, beyond which the compensated resistant mutants gain a competitive advantage and spread widely through the population (Figure 3d), thereby increasing the final size of the outbreak. The treatment level at which this minimum is achieved will be referred to as the optimal constant level. Although this pattern is qualitatively preserved for different reproduction numbers, the optimal treatment level is lower for smaller 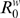 (Figure 3a), and therefore the outbreaks of drug-resistant infections become more likely even with moderate treatment levels. This suggests that reducing 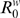 through application of other mitigation strategies may compromise the overall impact of antiviral therapy [9], should compensated mutants emerge (Figure 3d). However, in the absence of compensatory mutations, increasing treatment level would continue to decrease the epidemic size (Figure 3a, dashed curves), as resistant strains exist only at significantly lower transmission fitness compared with the wild-type strain. More importantly, the optimal level reduces as transmission fitness of the resistant strain exceeds a certain threshold and increases towards that of the wild-type virus. To demonstrate this, we performed sensitivity analyses over a range of key parameters, including the basic reproduction number of wild-type virus, the rates of de novo resistance, and the rates of conversion between resistant strains (see “Text S1”). Figure 4 displays the results of variations in the optimal treatment level as a function of δ*_rH_*. Although qualitatively similar results were obtained through a simple compartmental model [9], the integration of different stages of disease at the individual level and the likely delay in seeking healthcare and therefore initiating therapy, can have important consequences for developing pandemic plans [8].

### Adaptive treatment strategy

**Figure 5:**
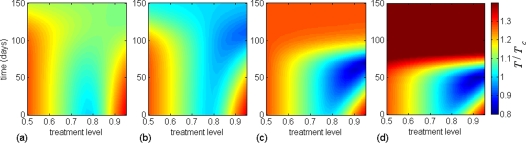
The effect of changing treatment level during a pandemic on the total number of clinical infections caused by all strains, with 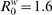. Simulations were seeded with an initial treatment level of (a) 85%; (b) 78% (optimal level); (c) 50%; (d) 25%, and then changed to the level shown on the horizontal axis at the time displayed on the vertical axis (corresponding to the time-course of the epidemic). The color bars illustrate the total number of clinical cases due to all strains, relative to that generated when the optimal constant treatment level (78% in this case) is implemented.

**Figure 6:**
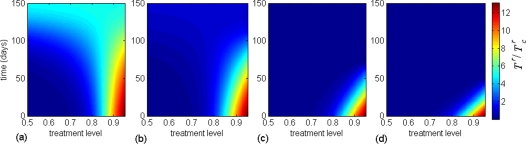
The effect of changing treatment level during a pandemic on the total number of clinical infections caused by the high fitness resistant strain, with 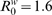. Simulations were seeded with an initial treatment level of (a) 85%; (b) 78% (optimal level); (c) 50%; (d) 25%, and then changed to the level shown on the horizontal axis at the time displayed on the vertical axis (corresponding to the time-course of the epidemic). The color bars illustrate the total number of clinical infections due to the resistant strain with high transmission fitness, relative to that generated when the optimal constant treatment level (78% in this case) is implemented.

In the event of emerging drug-resistance, it has been suggested [1] that reducing treatment levels may permit the wild-type to outcompete the resistant strains due to its greater fitness, thereby preventing large resistant outbreaks. To investigate this strategy, we modified the model to allow for changing the population−level of treatment at a specified time (*t* ^*^) during a pandemic. We define *T* as the total number of clinical infections when an antiviral strategy with varying level of treatment is implemented, and let *T_c_* be the total number of clinical infections when treatment is maintained at the optimal constant level. The ratio *T*/*T_c_* provides a criterion for identifying effective strategies for controlling the spread of disease. Assuming 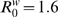, we simulated the model when antiviral treatment is initiated at an 85% level (above the corresponding optimal constant level 78%). The results show that the final size of infections may be reduced only if treatment is scaled down to the optimal level during the early stages of the pandemic (Figures 5a), before outbreaks of compensated mutants can occur (Figure 6a). Further simulations (Figure 5b) with a 78% initial treatment level indicate that reducing antiviral use, at any time during a pandemic, below the optimal level would increase the number of clinical infections (*T*/*T_c_*>1), suggesting that antiviral therapy at the optimal constant level will be more effective in mitigating a pandemic, and preventing resistant outbreaks (Figure 6b). However, the final size of a pandemic may be slightly reduced if the supply of drugs can afford a significant increase in the level of treatment at a later stage during the outbreak.

To further investigate the effect of raising treatment level, we simulated the model when treatment is initially administered at 25% and 50% (below the optimal level). The results show a significant reduction in the total number of clinical infections compared with that achieved at the optimal constant level (Figure 5c,d). However, the effectiveness of this strategy depends critically on the initial scale of drug-use and the time at which the level of treatment is raised. As is evident from Figure 5d, for lower treatment levels, an earlier increase in antiviral use is required for achieving the minimum final size. This is due to the fact that the wild-type virus spreads more rapidly (and therefore depletes the pool of susceptible individuals more quickly) during the initial low treatment phase. The findings suggest that the impact of this strategy is much more pronounced in mitigating a pandemic than a constant treatment plan, even if treatment can be maintained at the optimal level throughout the entire course of an outbreak. Figures 6c and 6d indicate that a timely increase in the level of drug-use can also prevent large outbreaks caused by the emergence of highly transmissible resistant viruses.

**Figure 7:**
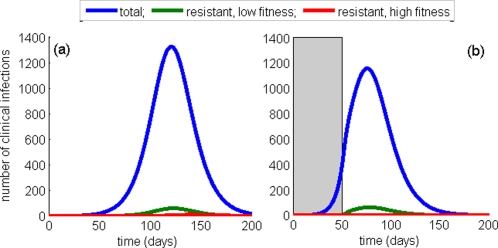
Time-courses of clinical infections with one day delay in onset of treatment of indexed cases for 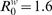. Simulations were run, when a single case infected with the wild-type virus is introduced into the susceptible population of size *S*_0_ = 100 000. Treatment in (a) is maintained at the optimal level (78%) for the entire course of the outbreak. Treatment in (b) is initiated at a 25% level for the first 50 days (shaded area), and then increased to 90% for the rest of the outbreak. All other parameters are the same as those used for simulations in Figure 2.

To further exemplify the overall benefit of this strategy, we simulated the time-courses of infection for two scenarios in which (i) a constant treatment level of 78% (optimal level) is administered at all times during the outbreak (Figure 7a); (ii) treatment is initiated at a 25% level for the first 50 days, and then increased to 90% for the rest of the outbreak (Figure 7b). In the second scenario, not only are clinical infections reduced, but also the spread of highly transmissible resistant viruses is prevented. In this scenario, the duration of the outbreak is shortened, with an earlier peak of infection. Compared with Figure 2c, in which treatment is kept at 90% from the beginning of the outbreak, it is observed that conservative treatment levels early on in a pandemic may be crucial in controlling the spread of resistant viruses. We also observed that even if the treatment level is only increased to 78% at day 50, from the initial 25% level, the small outbreak of the highly transmissible resistant strain shown in Figure 6a can be prevented, while the total number of clinical infections is also slightly reduced. Our sensitivity analyses, detailed in “Text S1”, show that these results remain robust across wide ranges of parameters that govern de novo resistance, compensatory mutations, and transmissibility of wild-type and resistant strains.

## Discussion

In this study, we extended a previous model for the emergence of drug-resistance [8] to evaluate the likely evolutionary-epidemiological outcomes of an antiviral treatment strategy. We discussed the influence of three major factors on the population-wide spread of drug-resistance, namely: (i) the reproduction number of the wild-type strain; (ii) time-dependent antiviral treatment level; and (iii) compensatory mutations that raise the replication fitness of resistant strains.

Our results show that, in the absence of compensatory mutations, resistant strains with large fitness cost cannot gain a competitive advantage in the spread of disease, and therefore increasing the level of antiviral treatment would reduce the final size of the pandemic (Figure 3a, dashed curves). While intensive drug use may increase the number of emergent resistant cases during treatment [8], [9], the population incidence of drug-resistance is still limited due to a significantly lower reproduction number (transmission fitness) of resistant strains compared with the wild-type virus.

In the presence of compensatory mutations, however, the competitive interference between wild-type and resistant strains is more complex. Since transmission fitness of compensated mutants is generally lower than that of the wild-type virus, the spread of disease is reduced by increasing drug use to moderate levels (Figure 3a, solid curves). While the number of emergent resistant cases increases with higher treatment levels (Figure 3c), the overall decrease in epidemic size is due to a more pronounced reduction of the wild-type transmission. As the use of antiviral drugs exceeds the optimal level, the overall epidemic size begins to grow, since the reproduction number of compensated mutants now stands well above that of the wild-type virus. Such wide-spread use of drugs will largely block transmission of the wild-type infection and greatly enhance the spread of drug-resistant viral mutants (Figure 3d), which in turn will increase the final size of the pandemic (Figure 3a, solid curve). Time courses of wild-type and resistant infections in Figure 2 illustrate these dramatic changes in the profile of outbreaks for a particular value of the reproduction number 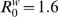. We observed similar behaviour for 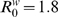 (Figure 3, black curves); however, the population-wide spread of drug-resistance requires more aggressive use of antiviral drugs.

While it is tempting to prescribe a high level of treatment at the onset of a pandemic for possible elimination of the wild-type virus, our simulations show that if aggressive treatment fails to contain the disease, then large outbreaks of resistant strains can develop. Considering a range of clinical attack rates above 25%, we have previously shown that an antiviral treatment as a single containment strategy will be unsuccessful at controlling the spread of wild-type disease if 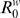 exceeds 1.4 [8]. Our findings in this study suggest that, as an alternative strategy, conservative treatment levels during the early stages of an outbreak can substantially contribute towards mitigating the pandemic burden. If followed by a timely increase in the level of drug-use, this strategy would preserve the potential for minimizing the final size of a pandemic (Figures 5c,d), while preventing large outbreaks of resistant viruses (Figures 6c,d). The principal mechanism underlying this adaptive antiviral strategy is to sufficiently reduce the number of susceptible individuals through an initial growth of wild-type infection, which will in turn prevent outbreaks of drug-resistant infections. However, an initial high treatment level followed by a reduction in antiviral use due to shortage in drug supply or emergence of highly transmissible drug-resistant strains in the population appears to be a poor strategy for disease control. We tested the robustness of our findings by performing sensitivity analyses over the estimated ranges of parameters describing the transmissibility of wild-type and resistant strains, de novo resistance emergence, and compensatory mutations that raise the fitness of resistant mutants. We also employed a previous population dynamical model for the emergence and spread of drug-resistance [9], and observed qualitatively consistent results for the proposed antiviral strategy. The findings of this study clearly indicate that any containment policy should be integrated with surveillance and monitoring systems, so that necessary adaptations to the treatment strategy can be made in a timely fashion, should resistant mutants with high transmission fitness emerge during the pandemic.

The modelling efforts in this study aim to evaluate the possible outcomes of various antiviral strategies. The work is meant to be a proof of concept rather than to provide specific quantitative recommendations for treatment policies, and we therefore emphasize the qualitative aspects of this evaluation. Nevertheless our evaluation, together with its sensitivity analyses, suggest that delaying implementation of aggressive treatment would reduce the overall disease burden, and significantly lower the probability of resistant outbreaks occurring. This strategy may be particularly beneficial when considering scarce resources of antiviral drugs, limited production capacity, and the surge in demand for treatment with the progression of a pandemic. Historical precedent, from both seasonal influenza epidemics and the 1918–1919 influenza pandemic, suggests that a novel influenza strain with high pathogenicity would severely tax existing health resources, and would force healthcare administrators and providers alike to make difficult decisions that may include rationing of scarce resources (e.g., antiviral drugs). Comparison of the potential consequences of competing strategies, as well as the practices required to achieve best outcomes, will allow for optimal resource allocation and health policy decisions.

For the results reported here, we assumed that antiviral drugs are used for treatment of only indexed cases having the same estimated efficacy of application as during interpandemic influenza outbreaks. However, the effect of antiviral therapy on the development of drug-resistance is much more pronounced when prophylactic use of drugs is planned in addition to treatment, as discussed in recent studies [9], [11]. Our simulations are based on parameters extracted from the published literature, and involve some degree of uncertainty, particularly with regard to the parameters that govern de novo resistance emergence and compensatory mutations. While highlighting the qualitative aspects of our study, parameter estimates from in vivo data associated with resistant mutations are needed to provide more accurate quantitative predictions. Combined with the previous work [8] that integrates the latest insights concerning within-host viral dynamics with the between-host spread of disease, a predictive framework of the emergence of drug-resistance is now on the horizon.

## Supporting Information

Text S1 Supplementary Material
(0.08 MB PDF)
