# Supplementary material for: Population-Wide Emergence of Antiviral Resistance during Pandemic Influenza
Source: PLoS One. 2008 Mar 19;3(3):e1839. doi: 10.1371/journal.pone.0001839 (PMC2266801; doi:10.1371/journal.pone.0001839)
Supplement: Text S1 — Supplementary Material (0.08 MB PDF) [file pone.0001839.s001.pdf]

Supplementary Information:
Population-wide emergence of antiviral resistance during pandemic influenza

Seyed M. Moghadas, Christopher S. Bowman, Gergely Röst, Jianhong Wu

This supplementary material details the development of the model and the derivation of the reproduction numbers associated with the wild-type and resistant strains. Model assumptions are described and estimates of parameters from published literature are given in Table 1. A sensitivity analysis was performed to ensure the robustness of the model's predictions over a range of key parameters, and the results are presented.[1] Further details of the model equations with regard to the within-host aspects of drug-resistance and integration with the between-host spread of disease can be found in (Alexander *et al.*, 2007).

1 The Model Structure

We assume that the population is entirely susceptible to the emerging pandemic strain with no pre-existing immunity. Let S denote the class of susceptible individuals who may become infected with either wild-type or resistant strains. Denoting the classes of individuals exposed to wild-type viruses by E, resistant strains with low fitness by E_r, and resistant strains with high fitness by E_{rH}, we have

$$
\begin{aligned}
S'(t) &= -\beta Q(t)S(t), \\
E'(t) &= \beta Q_w(t)S(t) - \mu_E E(t), \\
E_r'(t) &= \beta Q_r(t)S(t) - \mu_E E_r(t), \\
E_{rH}'(t) &= \beta Q_{rH}(t)S(t) - \mu_E E_{rH}(t),
\end{aligned}
\tag{1}
$$

where β is the baseline transmission rate of the wild-type strain, $1/\mu_E$ represents the mean latent period (assumed to be the same for wild-type and resistant infections), and $\beta Q(t) = \beta(Q_w + Q_r + Q_{rH})$ is the force of infection, yet to be formulated. Let p represent the probability of developing clinical disease after the latent period. Then, for corresponding classes of individuals with asymptomatic infections (i.e. those who are infectious without showing clinical symptoms, and therefore are not treated), we obtain

$$
\begin{aligned}
A'(t) &= (1-p)\mu_E E(t) - \mu_A A(t), \\
A_r'(t) &= (1-p)\mu_E E_r(t) - \mu_A A_r(t), \\
A_{rH}'(t) &= (1-p)\mu_E E_{rH}(t) - \mu_A A_{rH}(t),
\end{aligned}
\tag{2}
$$

[1]Simulations and sensitivity analyses of the model were performed using a solver for delay integro-differential equations (Paul, 1997).

Table 1: Description of the model parameters with their estimated values from the published literature (Ferguson *et al.*, 2005, 2006, 2003; Halloran *et al.*, 2006; Jefferson *et al.*, 2006; Longini *et al.*, 2004, 2005; Regoes & Bonhoeffer, 2006).

Parameter	Description	Value
$1/\mu_E$	mean latent period	1.25 days
$1/\mu_U$	mean infectious period of untreated symptomatic infection (secondary stage)	2.85 days
$1/\mu_T$	mean infectious period of treated symptomatic infection (secondary stage)	2.85 days
$1/\mu_A$	mean infectious period of asymptomatic infection	4.1 days
τ	mean infectious period of pre-symptomatic infection	0.25 day
n	duration of the window of opportunity for initiating antiviral treatment	2 days
δ_P	relative infectiousness of pre-symptomatic infection	0.286
δ_A	relative infectiousness of asymptomatic infection	0.142
δ_U	relative infectiousness of untreated symptomatic infection (secondary stage)	0.143
δ_T	relative infectiousness of treated symptomatic infection	0.4
d_U	death rate of untreated symptomatic infection with wild-type strain	0.002 day^{-1}
d_T	death rate of treated symptomatic infection with wild-type strain	0.0002 day^{-1}
$d_{U,r}$	death rate of symptomatic infection with low fitness resistant strain	0.0004 day^{-1}
$d_{U,rH}$	death rate of symptomatic infection with high fitness resistant strain	0.0016 day^{-1}
p	probability of developing clinical disease	0.67
V	fraction of treated individuals which develops resistance with low fitness	variable

where $1/\mu_A$ is the infectiousness period. To derive the equations for the symptomatic infection, we assume a window of opportunity of two days for start of treatment following the onset of clinical symptoms. Using rates of treatment and emergence of drug-resistance described in Alexander *et al.* (2007), the corresponding equations for untreated and treated symptomatic infections are given by

$$
\begin{aligned}
I'_U(t) =& p\mu_E E(t-n)q - (\mu_U + d_U)I_U(t),\\
I'_{U,r}(t) =& p\mu_E E_r(t-n)q - (\mu_U + d_{U,r} + \gamma_U)I_{U,r}(t),\\
I'_{U,rH}(t) =& p\mu_E E_{rH}(t-n)q + \gamma_U I_{U,r} - (\mu_U + d_{U,rH})I_{U,rH}(t),\\
I'_T(t) =& p\mu_E E(t-n)(1-q) - p\mu_E E(t-n)V - (\mu_T + d_T + \alpha_T)I_T(t),\\
I'_{T,r}(t) =& p\mu_E E_r(t-n)(1-q) + p\mu_E E(t-n)V + \alpha_T I_T - (\mu_U + d_{U,r} + \gamma_T)I_{T,r}(t),\\
I'_{T,rH}(t) =& p\mu_E E_{rH}(t-n)(1-q) + \gamma_T I_{T,r} - (\mu_U + d_{U,rH})I_{T,rH}(t),
\end{aligned}
\tag{3}
$$

where n is the size of the window of opportunity for the start of treatment; $1-q$ represents the population-level of treatment, μ_U and μ_T are the recovery rates of untreated and treated symptomatic infections (during secondary stage), respectively; d_U, $d_{U,r}$, and $d_{U,rH}$ are the corresponding disease-induced mortality rates for untreated symptomatic infections; V represents the fraction of treated individuals which develops drug-resistance with low fitness during the window of opportunity; α_T is the rate for developing drug-resistance during the secondary stage of symptomatic infection; and γ_U and γ_T are the rates of conversion between resistant mutants of untreated and treated symptomatic infections, respectively.

To formulate the force of infection, let $i_U(t,a)$ and $i_T(t,a)$ be the densities of untreated and treated wild-type infections after a time a has elapsed since an exposed individual becomes infectious. Consider-

ing the infectious compartments of the wild-type strain, and detailed description presented in Alexander *et al.* (2007, 2008), we have

$$Q_w(t) = \delta_A A(t) \qquad \text{(asymptomatic infection)}$$

$$+\delta_P \int_0^\tau i_U(t,a)da \qquad \text{(pre-symptomatic infection)}$$

$$+\int_\tau^n i_U(t,a) \qquad \text{(primary stage of symptomatic infection without treatment)}$$

$$+\delta_T \int_\tau^n i_T(t,a)da \qquad \text{(primary stage of symptomatic infection with treatment)}$$

$$+\delta_U I_U(t) \qquad \text{(secondary stage of symptomatic infection without treatment)}$$

$$+\delta_T \delta_U I_T(t) \qquad \text{(secondary stage of symptomatic infection with treatment)}$$

where τ is the period of pre-symptomatic infection; δ_A, δ_P, and δ_U represent the relative infectiousness of the wild-type strain for asymptomatic, pre-symptomatic, and the secondary stage of symptomatic infection without treatment, respectively; and δ_T is the relative infectiousness of a treated clinical case with the wild-type strain. The treatment is assumed to have no effect on individuals infected with resistant strains. With the corresponding notation for resistant strains, we have

$$Q_r(t) = \delta_r \delta_A A_r(t) \qquad \text{(asymptomatic infection)}$$

$$+\delta_r \delta_P \int_0^\tau i_{U,r}(t,a)da \qquad \text{(pre-symptomatic infection)}$$

$$+\delta_r \int_\tau^n i_{U,r}(t,a)da \qquad \text{(primary stage of symptomatic infection without treatment)}$$

$$+\delta_r \int_\tau^n i_{T,r}(t,a)da \qquad \text{(primary stage of symptomatic infection with treatment)}$$

$$+\delta_r \delta_U I_{U,r}(t) \qquad \text{(secondary stage of symptomatic infection without treatment)}$$

$$+\delta_r \delta_U I_{T,r}(t) \qquad \text{(secondary stage of symptomatic infection with treatment)}$$

and

$$
\begin{aligned}
Q_{rH}(t) = {} & \delta_{rH}\delta_A A_{rH}(t) && \text{(asymptomatic infection)} \\
& + \delta_{rH}\int_0^{\tau}\delta_P i_{U,rH}(t,a)da && \text{(pre-symptomatic infection)} \\
& + \delta_{rH}\int_{\tau}^{n} i_{U,rH}(t,a)da && \text{(primary stage of symptomatic infection without treatment)} \\
& + \delta_{rH}\int_{\tau}^{n} i_{T,rH}(t,a)da && \text{(primary stage of symptomatic infection with treatment)} \\
& + \delta_{rH}\delta_U I_{U,rH}(t) && \text{(secondary stage of symptomatic infection without treatment)} \\
& + \delta_{rH}\delta_U I_{T,rH}(t) && \text{(secondary stage of symptomatic infection with treatment)}
\end{aligned}
$$

where δ_r and δ_{rH} represent the relative infectiousness of resistant strains with low and high transmission fitness, respectively. It is assumed that treatment of wild-type infection reduces transmissibility of the virus by 60% (through reduction factor δ_T), but has no effect in transmission of resistant strains. Summarizing, the above represents the model as a system of delay differential equations, where estimates of its parameter values from the published literature are given in Table 1 (see also Table 1 in the main text).

2 Analysis of Reproduction Numbers

In our analysis, we fixed the initial size S_0 of the susceptible population to compute the control reproduction number R_C^w when an individual infected with the wild-type strain is introduced into the E-class. We assumed that $E(0) = 1$, and let $E(t) = 0$ for $t \in [-n, 0)$, and $A(0) = I_U(0) = I_T(0) = 0$. Considering the duration and transmission rates associated with asymptomatic, untreated and treated symptomatic infections (see Figure 1 in the main text), the total number of secondary cases generated in the A, I_U and I_T classes is given by

$$
\beta S_0\Big(\frac{(1-p)\delta_A}{\mu_A} + \frac{pq\delta_U}{\mu_U + d_U} + \frac{p(1-q-V)\delta_U\delta_T}{\mu_T + d_T + \alpha_T}\Big).
$$

We also calculated the number of new cases generated during the primary stage of symptomatic infection (window of opportunity for effective treatment), which involves the history of the E-class. Noting that $q = 1$ during pre-symptomatic infection (without treatment), this number is given by

$$
\begin{aligned}
&\int_0^{\infty}\beta S_0\Big[\int_0^{\tau}\delta_P i_U(t,a)da + \int_{\tau}^{n} i_U(t,a) + \delta_T i_T(t,a))da\Big]dt \\
&\quad = \beta S_0 p\Big(\delta_P\tau + \int_{\tau}^{n} q(a)da + \delta_T\int_{\tau}^{n}(1 - V(a) - q(a))da\Big),
\end{aligned}
$$

and therefore the control reproduction number of the wild-type strain can be expressed as

$$R_c^w = \beta S_0 \Big(\frac{(1-p)\delta_A}{\mu_A} + \frac{pq\delta_U}{\mu_U + d_U} + \frac{p(1-q-V)\delta_U \delta_T}{\mu_T + d_T + \alpha_T} + p\delta_P \tau + p(1-\delta_T)\int_\tau^n q(a)da + p\delta_T \int_\tau^n (1-V(a))da \Big).$$

In the absence of antiviral treatment ($q \equiv 1$ and $V \equiv 0$), R_c^w reduces to the basic reproduction number

$$R_0^w = \beta S_0 \Big(\frac{(1-p)\delta_A}{\mu_A} + \frac{p\delta_U}{\mu_U + d_U} + p\delta_P \tau + p(n-\tau) \Big).$$

Since treatment has no effect on individuals infected with resistant strains, similar calculations to the above lead to the reproduction numbers of resistant strains with low fitness (R_0^r) and high fitness (R_0^{rH}) as

$$R_0^r = \delta_r \beta S_0 \Big(\frac{(1-p)\delta_A}{\mu_A} + \frac{pq\delta_U}{\mu_U + d_{U,r} + \gamma_U} + \frac{p(1-q)\delta_U}{\mu_U + d_{U,r} + \gamma_T} + p\delta_P \tau + p(n-\tau) \Big),$$

and

$$R_0^{rH} = \delta_{rH} \beta S_0 \Big(\frac{(1-p)\delta_A}{\mu_A} + \frac{p\delta_U}{\mu_U + d_{U,rH}} + p\delta_P \tau + p(n-\tau) \Big).$$

The next generation matrix has the form

$$J = \begin{pmatrix} R_c^w & * & * \\ 0 & R_0^r & * \\ 0 & 0 & R_0^{rH} \end{pmatrix},$$

and therefore the criterion for the control of disease, defined in terms of the spectrum of this matrix (Diekmann & Heesterbeek, 2000), is given by $R_c = \max\{R_c^w, R_0^r, R_0^{rH}\}$.

3 Sensitivity Analysis

To investigate the effect of parameter changes on the results shown by simulations using baseline values, we performed a sensitivity analysis by considering a sampling approach that allows for the simultaneous variations of the basic reproduction number, the rates of de novo resistant mutations, and the rates of conversion between resistant strains. Using the Latin Hypercube Sampling (LHS) technique (McKay *et al.*, 1979), we generated samples of size n=100 in which each parameter is treated as a random variable and assigned a probability function. In this technique, the parameters are uniformly distributed and sampled within their respective ranges. The reproduction number R_0 was uniformly sampled from the range $[1.4, 2]$, which includes the estimated ranges of reproduction numbers for the 1918, 1957, and 1968 pandemics (Gani *et al.*, 2005; Viboud *et al.*, 2006). The rates of de novo resistant mutations ($\rho_{\max}$, α_T) were sampled from the range $[0.018, 0.072]$ (Regoes & Bonhoeffer, 2006; Débarre *et al.*, 2007), corresponding to $5.8\% - 17\%$ incidence of resistance, which lies within the estimated range of neuraminidase resistance reported in clinical studies (Kiso *et al.*, 2004; Ward *et al.*, 2005; Yen *et al.*, 2005). The corresponding ranges for the conversion rates of resistant strains (γ_U, γ_T) was computed using the constraint that the fraction of treated individuals hosting resistance, which undergoes compensatory mutations and subsequently generates resistant strains with high fitness, lies between $1/5000$ and $1/500$ (Lipsitch *et al.*, 2007). The baseline value of 1/2000 was used for simulations. Furthermore, we assumed

that compensatory mutations are less likely to occur in the absence of treatment, and considered γ_U to be $10-$fold smaller than γ_T (Handel *et al.*, 2006). The values of other parameters are given in Table 1.

For the sensitivity analyses, we introduced the parameter T_a to represent the “minimum final size” of the pandemic within an adaptive treatment strategy, when the initial treatment level is increased to 90% at a time t^*. For each set of parameter values in the sample, we then computed the ratio T_a/T_c as a function of δ_{rH} (the relative transmission of the resistant strain with high fitness), where T_c is the final size of the pandemic when treatment is maintained constant at the corresponding optimal level (below 90%) at all times during the outbreak. The results of sensitivity analyses are illustrated in Figures 1a, 1b, 1c, when initial treatment levels in the adaptive treatment strategy are assumed to be 0%, 25%, and 50%, respectively. These figures indicate that for low values of δ_{rH} (below ~ 0.8), the risk of a resistant epidemic developing is small, and both treatment strategies are comparable in their effectiveness. However, as δ_{rH} increases (above ~ 0.8), self-sustaining epidemics of resistant viruses can be established, and the benefit of an adaptive treatment strategy becomes more pronounced. Using the above sample, we also projected the corresponding ranges of time t^* as a critical parameter in this strategy. The results, depicted in Figures 2a, 2b, 2c, suggest that aggressive treatment should be further delayed (following the onset of the outbreak) for higher initial treatment levels, should resistant strains with high transmission fitness (above ~ 0.8) emerge. Such high treatment levels decelerate the spread of the wild-type virus in the population, and therefore extend the time required for a sufficient drop in the number of susceptible individuals to prevent resistant outbreaks.

(a)

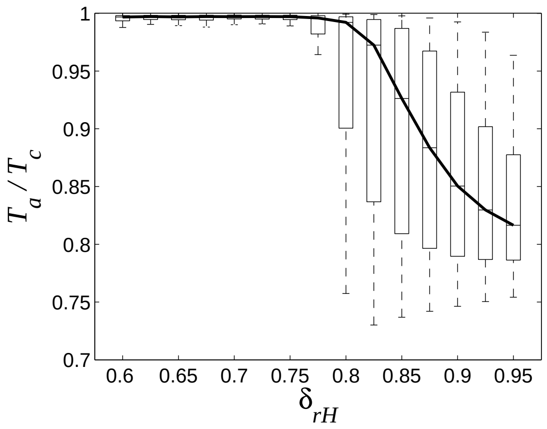

(b)

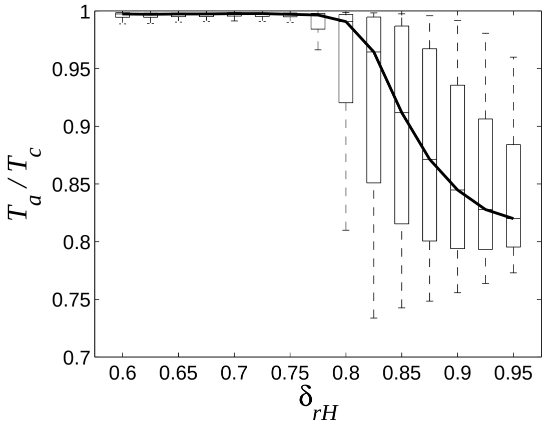

(c)

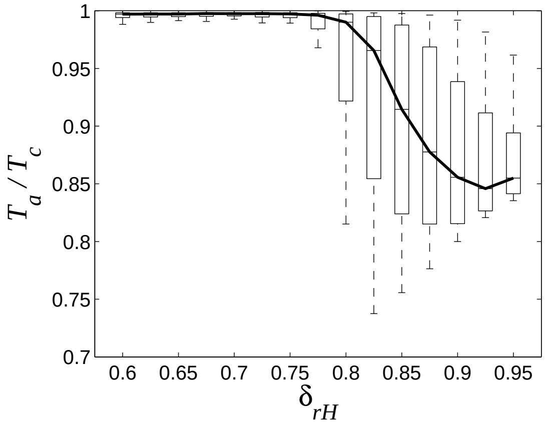

Figure 1: Sensitivity analyses showing box plots for the variations in the ratio T_a/T_c as a function of δ_{rH}, with other parameters sampled from their respective ranges, as described in the text. The solid curve passes through the median values of the ratio T_a/T_c, and each box contains 50% of data points between the first and third quartiles of the sampling distribution. The remaining 50% of data points are represented by whiskers. Initial treatment levels in the adaptive antiviral strategy before transition time t^* are: (a) 0%; (b) 25%; and (c) 50%.

(a)

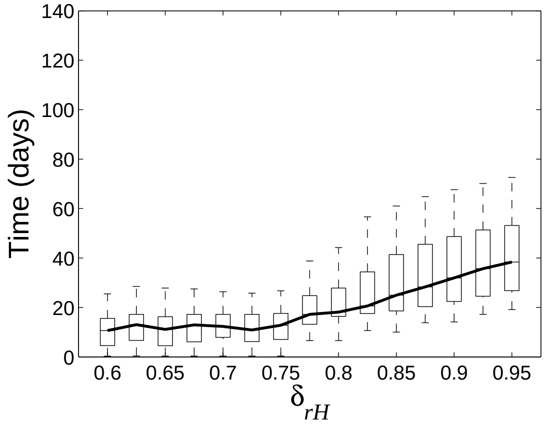

(b)

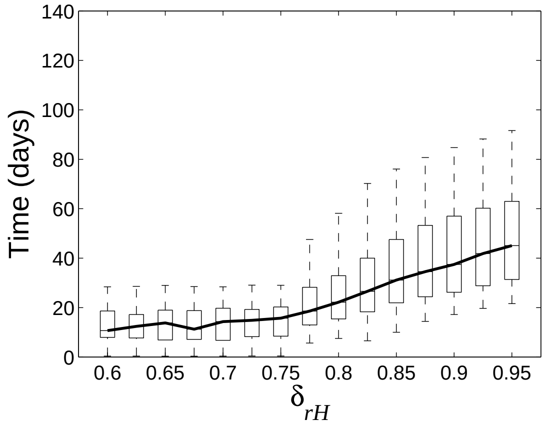

(c)

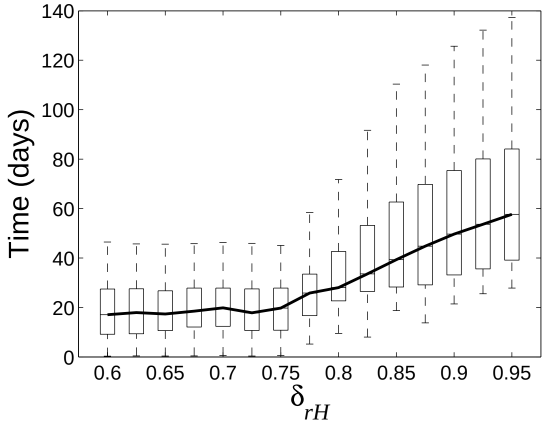

Figure 2: Sensitivity analyses showing box plots for the variations in the optimal transition time t^* corresponding to the minimum total number of infections, as a function of δ_{rH}, with other parameters sampled from their respective ranges, as described in the text. The solid curve passes through the median values of data points for t^*, and each box contains 50% of data points between the first and third quartiles of the sampling distribution. The remaining 50% of data points are represented by whiskers. Initial treatment levels in adaptive antiviral strategy before transition time t^* are: (a) 0%; (b) 25%; and (c) 50%.

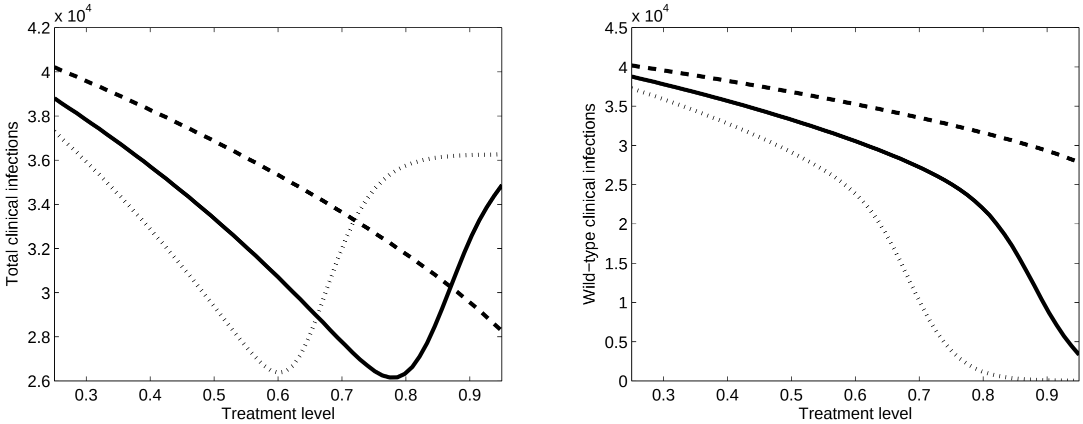

Figure 3: (a) Total number of clinical infections caused by all strains; and (b) Total number of wild-type clinical infections, as a function of treatment level, with $R_0^w = 1.6$. Dotted, solid, and dashed curves correspond respectively to 0.5, 1, and 1.5 days delay in initiating treatment after the onset of clinical disease.

4 The Effect of Delay in Start of Treatment

Not only the population level of drug use, but also early onset of treatment of indexed cases within the window of opportunity can significantly influence the outcome of an antiviral strategy. To demonstrate this, we compared the final size of clinical infections in three scenarios with different delays in the start of treatment following the onset of clinical disease. Figure 3a (dotted curve) shows that early treatment with 0.5-day delay results in smaller number of clinical infections (and therefore the minimum epidemic size is feasible with a lower level of drug use) than when treatment is initiated with 1-day delay (solid curve). This is mostly due to a greater reduction in transmission of the wild-type infection (Figure 3b). A more rapid decline in the final size of wild-type infections occurs when compensated mutants become the driving force for disease progression with increasing level of treatment. However, a more dramatic increase in the number of resistant infections is observed for higher levels of drug use with less delay in start of treatment (Figure 3a, dotted curve). Initiating treatment with a longer delay of 1.5 days has little impact on suppressing wild-type infection, even with high levels of treatment (Figure 3b, dashed curve). However, in this case, resistance emergence is limited due to the wide spread of the wild-type virus, thereby rapidly depleting the pool of susceptible hosts.

References

Alexander ME, Bowman CS, Feng Z, Gardam M, Moghadas SM, et al (2007) Emergence of drug-resistance: implications for antiviral control of pandemic influenza, Proc. R. Soc. B 274: 1675–1684.

Alexander ME, Moghadas SM, Röst G, Wu J (2008) A delay differential model for pandemic influenza with antiviral treatment, Bull. Math. Biol. 70: 382–397.

Débarre F, Bonhoeffer S, Regoes RR (2007) The effect of population structure on the emergence of drug-resistance during pandemic influenza, J. R. Soc. Interface 4: 893–906.

Diekmann O, Heesterbeek JAP (2000) Mathematical Epidemiology of Infectious Diseases, Wiley, Chichester.

Ferguson NM, Cummings DAT, Cauchemez S, Fraser C, Riley S, et al (2005) Strategies for containing an emerging influenza pandemic in Southeast Asia, Nature 437: 209–214.

Ferguson NM, Cummings, DAT, Fraser C, Cajka JC, Cooley PC, et al (2006) Strategies for mitigating an influenza pandemic, Nature 442: 448–452

Ferguson NM, Mallett S, Jackson H, Roberts N, Ward P (2003) A population-dynamic model for evaluating the potential spread of drug-resistant influenza virus infections during community-based use of antivirals, J. Antimicrob. Chemother. 51: 977–990.

Gani R, Hughes H, Fleming D, Griffin T, Medlock J, et al (2005) Potential impact of antiviral drug use during influenza pandemic, Emerg. Infect. Dis. 9: 1355–1362.

Halloran ME, Hayden FG, Yang Y, Longini Jr. IM, Monto AS (2006) Antiviral effects on influenza viral transmission and pathogenicity: observations from household-based trials, Am. J. Epidemiol. DOI:10.1093/aje/kwj362.

Handel A, Regoes, RR, Antia R (2006) The role of compensatory mutations in the emergence of drug resistance, PLoS Comput. Biol. 2: 1262–1270.

Jefferson T, Demicheli V, Rivetti D, Jones M, Di Pietrantonj C, et al (2006), Antivirals for influenza in healthy adults: systematic review, Lancet 367, 303-313.

Kiso M, Mitamura K, Sakai-Tagawa Y, Shiraishi K, Kawakami C, et al (2004) Resistant influenza A viruses in children treated with oseltamivir: descriptive study, Lancet 364: 759–765.

Lipsitch M, Cohen T, Murray M, Levin BR (2007) Antiviral resistance and the control of pandemic influenza, PLoS Med. 4 e15: 0111–0121.

Longini Jr. IM, Halloran ME, Nizam A, Yang Y (2004) Containing pandemic influenza with antiviral agents, Am. J. Epidemiol. 159: 623–633.

Longini Jr. IM, Nizam A, Xu S, Ungchusak K, Hanshaoworakul W, et al (2005) Containing pandemic influenza at the source, Science 309: 1083–1087.

McKay MD, Conover WJ, Beckman RJ (1979) A comparison of three methods for selecting values of input variables in the analysis of output from a computer code, Technometrics 21: 239-245.

Paul CAH (1997) A user-guide to ARCHI-an explicit Runge-Kutta code for solving delay and neutral differential equations and parameter estimation problems, Technical Report 283, Department of Mathematics, University of Manchester.

Regoes RR, Bonhoeffer S (2006) Emergence of drug-resistance influenza virus: population dynamical considerations, Science 312: 389–391.

Viboud C, Tam T, Fleming D, Handel A, Miller MA, et al (2006) Transmissibility and mortality impact of epidemic and pandemic influenza, with emphasis on the unusually deadly 1951 epidemic, Vaccine 24: 6701–6707.

Ward P, Small I, Smith J, Suter P, Dutkowshi R (2005) Oseltamivir (Tamiflu©) and its potential for use in the event of an influenza pandemic, Antimicrob. Agents Chemother. 55: Suppl. S1, i5–i21.

Yen HL, Herlocher LM, Hoffmann E, Matrosovich MN, Monto AS, et al (2005) Neuraminidase inhibitor-resistant influenza viruses may differ substantially in fitness and transmissibility, Antimicrob. Agents Chemother. 49: 4075–4084.
